# Effects of Substituents on the Blue Luminescence of Disilane-Linked Donor‒Acceptor‒Donor Triads

**DOI:** 10.3390/molecules24030521

**Published:** 2019-01-31

**Authors:** Tsukasa Usuki, Kenichiro Omoto, Masaki Shimada, Yoshinori Yamanoi, Hidetaka Kasai, Eiji Nishibori, Hiroshi Nishihara

**Affiliations:** 1 Department of Chemistry, School of Science, The University of Tokyo, 7-3-1 Hongo, Bunkyo-ku, Tokyo 113-0033, Japan; usuki@chem.s.u-tokyo.ac.jp (T.U.); omoto@chem.s.u-tokyo.ac.jp (K.O.); s.masaki14@gmail.com (M.S.); 2 Division of Physics, Faculty of Pure and Applied Sciences, Tsukuba Research Center for Interdisciplinary Materials Science (TIMS), and Center for Integrated Research in Fundamental Science and Engineering (CiRfSE), University of Tsukuba, 1-1-1 Tennodai, Tsukuba, Ibaraki 305-8571, Japan; kasai.hidetaka.fw@u.tsukuba.ac.jp (H.K.); nishibori.eiji.ga@u.tsukuba.ac.jp (E.N.)

**Keywords:** disilane, donor‒acceptor, optical properties, solid-state emission, X-ray diffraction, DFT calculation

## Abstract

A series of disilane-linked donor‒acceptor‒donor triads (D‒Si‒Si‒A‒Si‒Si‒D) was synthesized to investigate the effects of substituents on the photophysical properties. The triads were prepared by metal-catalyzed diiodosilylation of aryl iodides using a Pd(P(*t*-Bu)_3_)_2_/(*i*-Pr)_2_EtN/toluene system that we previously developed. Optical measurements, X-ray diffraction analysis, and density functional theory calculations revealed relationships between the photophysical properties and molecular structures of these triads in solution and in the solid state. The compounds emitted blue to green fluorescence in CH_2_Cl_2_ solution and in the solid state. Notably, compound **2** showed fluorescence with an absolute quantum yield of 0.17 in the solid state but showed no fluorescence in CH_2_Cl_2_. Our findings confirmed that the substituent adjacent to the disilane moiety affects the conformations and emission efficiencies of compounds in solution and in the solid state.

## 1. Introduction

Compounds in which electron-donating and electron-accepting groups are connected by π-conjugated linkers have recently attracted much attention as luminescent molecules [1,2]. These donor–acceptor (D/A) molecules exhibit photoabsorption and emission characteristics arising from intramolecular charge transfer (ICT) between the donor and acceptor. The luminescence properties of D/A molecules strongly depend on the type of donor and acceptor moieties, allowing their photophysical properties to be tuned precisely by combining appropriate donor and acceptor moieties. Thus, D/A molecules can be readily designed, but in the solid state, conventional D/A molecules suffer from emission quenching due to intermolecular π···π stacking. Consequently, in contrast to the large number of molecules that are highly emissive in solution, there are a few examples of light emissive organic solids with high fluorescence quantum yield [3,4,5].

To design compounds exhibiting intense solid-state emission, we recently focused our attention on organic silicon luminophores containing a σ‒π conjugated system comprising Si‒Si σ and C=C π bonds. In these σ‒π conjugated systems, the silicon atom can expand conjugation while maintaining its sp^3^ structure, and σ‒π conjugated molecules probably adopt spatially expanded structures [6,7,8,9,10]. The bulky structures of σ‒π conjugated organosilicon molecules can suppress intermolecular π···π stacking, thereby suppressing non-radiative decay to realize efficient solid-state emission.

We have previously developed various solid-state emissive organosilicon molecules [11,12,13,14]. For example, in D‒Si‒Si‒A‒Si‒Si‒D molecules, which are also called disilane-linked D/A/D molecules, two donor and acceptor moieties are alternately linked by disilane units. We found that disilane-linked D/A/D molecules are highly emissive even in the solid state due to steric hindrance of the disilane moieties, which suppresses π···π stacking [15,16]. These compounds exhibit photoabsorption and luminescence properties based on ICT between the donor and acceptor through the σ‒π conjugated system. Consequently, the optical properties of disilane-linked D/A/D molecules can be tuned based on the type of donor and acceptor moieties, similar to conventional π-conjugated D/A molecules. To achieve the precise design and prediction of the optical properties of disilane-linked D/A/D molecules, understandings of the relationship between structure and the photophysical properties of disilane-linked molecules are required.

**Figure 1:**
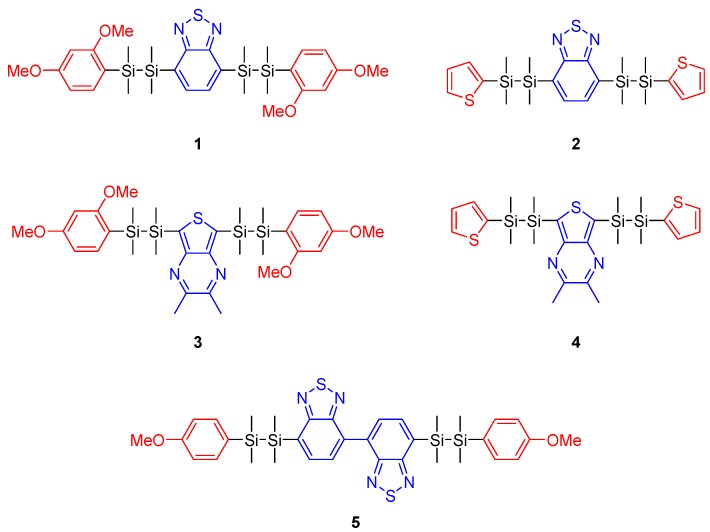
Chemical structures of disilane-bridged D/A/D molecules synthesized in this work. Red marked groups: aromatic electron-donating substituents. Blue marked groups: aromatic electron-accepting substituents.

In this work, we clarified the effects of substituents on the optical properties of disilane-linked D/A/D molecules. We have previously reported various disilane-linked D/A/D molecules, but the correlation between molecular structure and optical properties requires further clarification. Here, we designed and synthesized a series of new disilane-linked D/A/D molecules **1**–**5** (Figure 1) possessing benzothiadiazole or thienopyrazine cores as the electron-accepting groups. Additionally, two types of electron donors were investigated: compounds **1** and **3** possessed two 2,4-dimethoxyphenyl functionalities, and **2** and **4** possessed two thiophene functionalities covalently linked to the acceptor core through disilane units. Compound **5** comprised a strong electron-accepting bibenzothiadiazole functionality in the core, connected to Si‒Si linked *p*-methoxyphenyl rings at the termini. The effects of these functionalities on the optical properties of the compounds were studied by UV-vis absorption, photoemission and single-crystal X-ray diffraction (XRD) analyses, and computational calculation.

## 2. Results and Discussion

### 2.1. Synthesis

We synthesized disilane-linked donor–acceptor molecules **1**–**5** (Figure 1). The Pd-catalyzed coupling of 1-(2,4-dimethoxyphenyl)-1,1,2,2-tetramethyldisilane or 1-(2-thiophenyl)-1,1,2,2-tetramethyldisilane and diiodoarene, which has been previously reported by our group [14,15,16], afforded the target molecules in moderate yield without cleavage of the Si‒Si bond via deiodosilylation. The syntheses are described in detail in the Experimental section. The resulting products were characterized by high-resolution mass spectrometry (HRMS) and ^1^H and ^13^C-NMR spectroscopy. All the compounds are soluble in common organic solvents. Compounds **4** and **5** are viscous oils and compounds **1**, **2**, and **3** are colorless powders at room temperature.

### 2.2. Optical Properties of the Disilane-Bridged Compounds in Solution and in the Solid States

**Figure 2:**
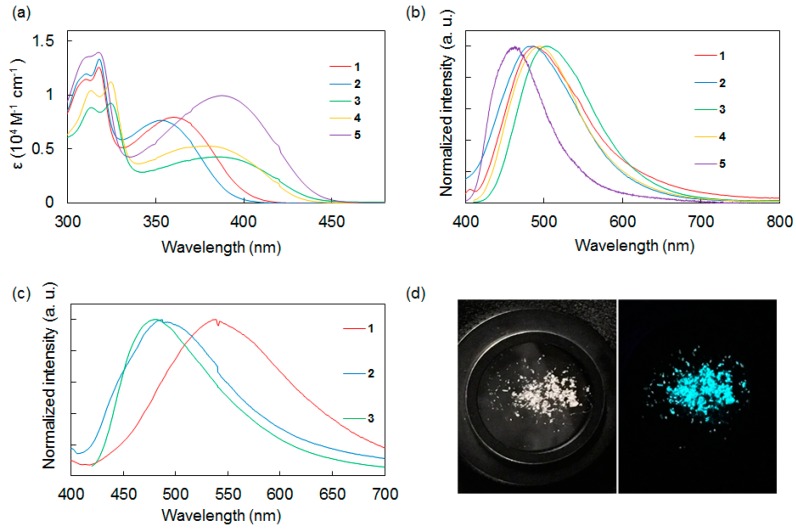
(**a**) UV-vis absorption spectra of **1**–**5** in CH_2_Cl_2_. (**b**) Photoluminescence spectra of **1**–**5** in CH_2_Cl_2_. (**c**) Photoluminescence spectra of **1**–**3** in the solid state. (**d**) Photographs of **2** in the solid state. Left: under ambient light. Right: under UV light (*λ*_ex_ = 365 nm).

With the synthesized molecules in hand, we conducted UV-vis absorption and fluorescence spectroscopic measurements to investigate the relationship between the structure and photophysical properties of the compounds. These results are shown in Table 1 and Figure 2. The UV-vis absorption spectra of **1**–**5** in CH_2_Cl_2_ were measured at room temperature. Compounds **1**–**5** showed broad absorption bands in the range of 350–390 nm with moderate molar extinction coefficients (*ε*: 4280–9960 M^−1^ cm^−1^), corresponding to local excitation on each aromatic ring (π→π*) and the charge transfer intramolecular interaction between the donor and acceptor moieties. 

The absorption spectra of the compounds strongly depended on the electron-accepting functional group. Compounds **1** and **2**, which had a benzothiadiazole acceptor, exhibited an absorption band around 360 nm. In contrast, **3** and **4**, which had a thienopyrazine group, exhibited a longer absorption wavelength around 380 nm. The absorption band of **5**, which has a bibenzothiadiazole core, exhibited a bathochromic shift relative to **1**–**4**. The effects of the acceptor cores on the shapes of the absorption spectra appeared to be stronger than that of the donor terminal groups, consistent with other disilane-linked molecules previously reported by our group [15,16,17,18,19].

Next, the photoluminescence properties of **1**–**5** in CH_2_Cl_2_ at room temperature were studied. In solution, compounds **1**–**5** emitted blue to green fluorescence (*λ*_max_ = 460–503 nm) based on ICT. Compound **5**, which had a bibenzothiadiazole acceptor, showed a shorter emission wavelength band and its Stokes shift was smaller than those of **1**–**4**. This result suggested that **5** underwent smaller structural relaxation in the excited state compared with **1**–**4**.

Comparison of **1**–**4** showed that the donor/acceptor combination strongly affected the emission intensity in CH_2_Cl_2_. The compounds with a thiophene donor or a benzothiadiazole acceptor showed weak fluorescence, whereas the compounds with a dimethoxybenzene donor or a thienopyrazine acceptor displayed stronger fluorescence. For example, compound **2**, which had thiophene and benzothiadiazole moieties, showed a low fluorescence quantum yield (*Ф*_F_ = 0.002), whereas compound **3**, which had a dimethoxybenzene donor and a thienopyrazine acceptor, exhibited a higher fluorescence quantum yield (*Ф*_F_ = 0.097).

We investigated the effects of donor moieties on emission efficiency in CH_2_Cl_2_ by comparing the rate constants of the photophysical processes of **3** and **4**. The fluorescence rate constants (*k*_F_) were similar value, although the non-radiative rate constant (*k*_nr_) of **3** was about one fifth that of **4**. This result suggested that some kind of intramolecular motion, such as internal rotation about the Si–Si or Si–C σ bonds, was suppressed by steric hindrance of the methoxy group at the *o*-position against to Si–Si σ bond.

The emission intensities of benzothiadiazole compounds **1** and **2** were too weak to estimate their fluorescence life time and rate constants, making it difficult to examine the effect of the acceptor moiety on emission efficiency in CH_2_Cl_2_. Despite the present lack of a detailed mechanism, the current results are consistent with our earlier finding that the emission intensity of disilane-linked D/A/D molecules with benzothiadiazole core is weak in solution [15,16].

The photoluminescence characteristics of compounds **1**–**3** in the solid state at room temperature were also investigated. Compounds **1**–**3** exhibited blue to green fluorescence, similar to that observed in solution, and their luminescence efficiencies were strongly affected by the substituents on the donor moieties. Similar to previously reported disilane-linked D/A/D molecules [15,16], thiophene compound **2** provided a high solid-state fluorescence quantum yield of *Ф*_F_ = 0.17. This value is nearly two orders of magnitude larger than the value obtained for **2** in solution (*Ф*_F_ = 0.002 in CH_2_Cl_2_), suggesting the suppression of intramolecular motion and intermolecular π···π stacking in the solid state, and the reduction of non-radiative quenching. Recently, Skorotetckey and Ponomarenko reported 4,7-Bis{4-[5-(trimethylsilyl)-2-thienyl]phenyl}-2,1,3-benzothiadiazole, which had the similar structure of **2**, showed decreased photoluminescence in the solid state in comparison with solution state [20]. The photoluminescence quenching may be due to H-aggregation in the solid state. In contrast, *o,p*-dimethoxybenzene compounds **1** and **3** showed low fluorescence quantum yields in the solid state, with the emission efficiency of **3** in the solid state being one fifth of that in solution. The specific molecular conformations of **1** and **3** in the solid state imposed by the bulkiness of the *o,p*-dimethoxybenzene moieties may affect the photophysical processes in these compounds substantially [21]. The correlation between emission intensity and the molecular structure of dimethoxybenzene compounds in the solid state is described below. 

### 2.3. XRD Analysis

**Figure 3:**
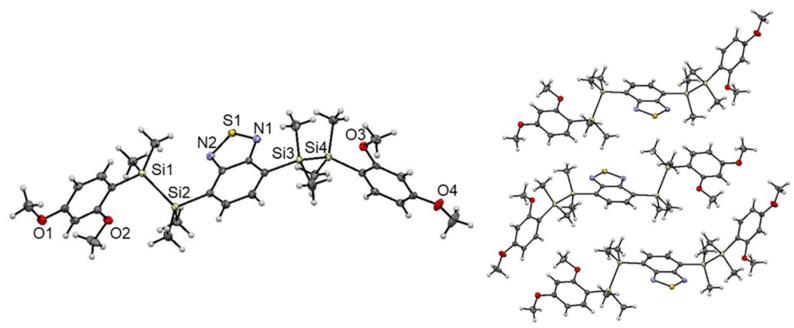
Crystal structure of **1** (**left**). Thermal ellipsoids represent 50% probability. Packing structure of **1** (**right**).

We investigated the molecular structure and packing of **1** in the solid state by single-crystal XRD analysis (Figure 3). Crystallization of **1** from methanol at 0 °C provided single crystals suitable for XRD analysis [22]. In the structure, the Si atoms adopt slightly distorted tetrahedral geometries with an average Si‒C bond length of approximately 1.88 Å. The Si‒Si distance (Si1‒Si2: 2.349(1) Å, Si3‒Si4: 2.355(1) Å) of **1** is comparable to that of known disilane compounds [23,24,25]. The central acceptor core and two donor terminal aryl groups are arranged in the *trans* configuration. The steric bulkiness of the disilane units efficiently suppresses π···π stacking in the solid state. Notably, the molecular structure of **1** adopted a *twisted* conformation, where π-planes of adjacent donor and acceptor moieties are *not* in parallel in each other and disilane bonds are *not* perpendicular to them, probably due to steric repulsion between the methyl groups on the silicon atoms and the MeO-group at the *o*-position of the donor moieties. Such twisted conformation of **1** is different from that of many other already reported disilane-linked D/A/D molecules, in which the aromatic rings of the donor/acceptor adopt a parallel arrangement [15,16]. Despite numerous trials, X-ray crystallographic analysis of **3** possessing *o,p*-dimethoxybenzene moieties was not successful. Assuming of the similarity the molecular structures to that of **1**, **3** is supposed to form a twisted structure in the solid state likewise **1**. The twisted conformation of **1** and **3** might reduce the intramolecular electronic interaction between the donor and acceptor moieties, thereby suppressing radiative decay and accelerating non-radiative decay, resulting in exceptionally low fluorescence quantum yields in the solid state [26].

We speculated crystal structure of **2** from powder XRD and Rietveld analysis (Materials and Methods, Figures S1 and S2). The structure can be predicted by the direct space strategy from PXRD (powder X-ray diffraction) pattern using the genetic algorithm. One of the plausible molecular structure and packing mode of **2** are basically similar to those of **1**, where the central acceptor core and two donor terminal aryl groups are arranged in the *trans* configuration without forming intermolecular π···π stacking. However, in contrast to **1**, molecular structure of **2** adopt a *step-like* configuration, where the π-plane of aromatic rings of the donor and acceptor moieties are in parallel and disilane bonds are perpendicular to them, likewise many other already reported disilane-linked D/A/D molecules [15,16]. These molecular structure and packing mode of **2** might affect the intramolecular electronic interaction between the donor and acceptor moieties, thereby suppressing the deactivation process to trigger the intense fluorescence in the solid state. 

### 2.4. Computational Investigation

**Figure 4:**
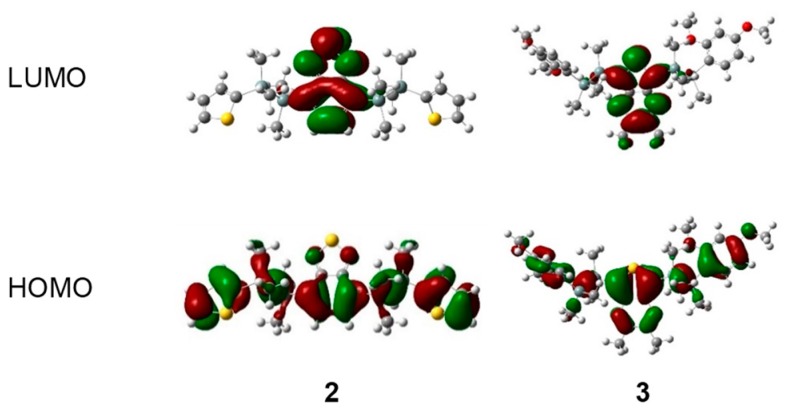
Electron density distribution of frontier molecular orbitals of representative compounds **2** and **3**, calculated at the B3LYP/6-31G (d, p) level.

We conducted density functional theory (DFT) and time-dependent density functional theory (TD-DFT) calculations to gain deeper insights into our results. These DFT and TD-DFT calculations were performed at the B3LYP/6-31G (d, p) level of theory [27]. Spatial distributions of the molecular orbitals of disilane-linked D/A/D molecules **1**–**5** are shown in Figure 4 and Figure S3. In the HOMO (highest occupied molecular orbital), electron density was distributed over the whole molecule, with contributions from the Si‒Si σ bonding orbitals, whereas in the LUMO (lowest unoccupied molecular orbital), the electron density was concentrated on the acceptor segment. TD-DFT calculations performed to explain the observed photophysical properties of **1**–**5** showed that the lowest-energy excitation was mainly attributed to the nature of the HOMO→LUMO transition with a high oscillator strength (Tables S3–S7). This result indicates that **1**–**5** adopted mixed excited states consisting of a charge transfer state and a local excited state on the acceptor core, further supporting our observations that the photoabsorption properties of **1**–**5** were substantially affected by the acceptor core.

## 3. Materials and Methods 

### 3.1. General Information

Unless otherwise indicated, all solvents and reagents were purchased from commercial sources and used without further purification. All synthetic experiments were performed under a nitrogen or argon atmosphere. Water was purified with a water purification system (AUTOPURE WD500, Yamato Scientific Co., Ltd., Tokyo, Japan). Dehydrated tetrahydrofuran, diethyl ether, and toluene were purchased from Kanto Chemical Co., Inc. (Tokyo, Japan). These solvents were purified using an organic solvent purifier (Nikko Hansen Co., Ltd., Osaka, Japan) and stored over 4 Å molecular sieves under argon prior to use. The starting materials, 4,7-diiodobenzo[c][1,2,5]thiadiazole, 5,7-diiodo-2,3-dimethylthieno[3,4-b]pyrazine, and 7,7′-diiodo-4,4′-dibenzo[c][1,2,5]thiadiazole, were prepared using literature protocols [28].

Melting points were obtained on a melting point apparatus (MP-S3, Yanako, Tokyo, Japan) and were uncorrected. NMR spectra were measured with an NMR spectrometer (US500, Bruker, Billerica, MA, USA). Tetramethylsilane (δ = 0.00) was used as an internal standard for the ^1^H-NMR spectra, and CDCl_3_ (δ = 77.0) was used as an internal standard for the ^13^C-NMR spectra. Gas chromatography-mass spectrometry (GC-MS) spectra were recorded on a GC-MS system (GC-MS-QP2010, Shimadzu, Tokyo, Japan). Low- and high-resolution FAB (fast atom bombardment) mass spectra were measured with a high-resolution mass spectrometer (JMS-700 MStation, JEOL, Tokyo, Japan). Absorption spectra were measured with a spectrophotometer (V-570, JASCO, Tokyo, Japan) using a 1 cm path length quartz cell. Fluorescence spectra were measured with a spectrofluorometer (FP-8600, JASCO, Tokyo, Japan). Fluorescence quantum yields were determined by the integrating sphere method on an absolute PL (photoluminescence) quantum yield measurement system (Hamamatsu C9920-01, Hamamatsu, Japan). Optical measurements were conducted using 10^−5^ M **1**–**5** in solution. Fluorescence lifetimes were measured with a Hamamatsu Quantaurus-Tau C11367-02 spectrometer (Hamamatsu, Japan). Preparative gel permeation liquid chromatography was performed using a liquid chromatography system (LC-92XXII NEXT SERIES, JASCO, Tokyo, Japan).

### 3.2. X-ray Crystallographic Analysis

Single crystals suitable for XRD analysis were obtained by crystallization from a methanol-saturated solution of **1** using the slow evaporation method at 277 K. Structural data for **1** are summarized in Table S1. Single-crystal XRD data were collected using dual optics (VariMax, Rigaku, Tokyo, Japan) with a diffractometer (Saturn 724+, Rigaku) and MoKα radiation (*λ* = 0.71075 Å) at 97 K. The structures were solved by direct methods using SHELXL-97 [29] and refined by full-matrix least squares methods on *F*^2^. All non-hydrogen atoms were refined anisotropically and all hydrogen atoms were placed in calculated positions and refined using idealized geometries (riding model) and assigned fixed isotropic displacement parameters.

### 3.3. Powder XRD Analysis

The powder crystals were installed in a 0.4 mm glass capillary. The synchrotron powder diffraction experiments of **2** with an Imaging Plate (IP) as a detector were carried out at SPring-8, BL02B2 beam line [30]. The powder data of **2** were collected at 100 K using N_2_ gas flow low temperature device. Indexing was carried out using the program DICVOL06 [31]. The first 20 peaks of the powder pattern were completely indexed on the basis of a monoclinic cell. The space group was uniquely determined as *P*n from the reflection conditions. The crystal structure of **2** has been predicted by the structure solution system based on genetic algorithm (GA) [32]. A Rigid-Body Rietveld analysis [33] was employed for the refinement. The reliability factor of the Rietveld refinement was *R*_wp_ = 6.4% with d > 0.97 Å d-spacing range.

### 3.4. Computational Details

DFT calculations were performed using Gaussian 09, Revision E.01. Molecules were optimized at the B3LYP/6-31G (d,p) level of theory and subsequent vibrational frequency calculations confirmed the absence of imaginary frequencies. The electronic structures at the excited state were predicted using TD-DFT formalism. Optoelectronic properties were calculated using the optoelectronics panel available in the Schrödinger Materials Science Suite 2017-166 using the B3LYP function and midix basis set, keeping all other parameters at their default values.

### 3.5. Typical Experimental Procedure for the Synthesis of ***1***–***5***

Under an argon atmosphere, *N,N*-diisopropylethylamine (0.78 mL, 4.0 mmol) and 1-(2,4-dimethoxyphenyl)-1,1,2,2-tetramethyldisilane (0.56 mL, 2.2 mmol) were added to a solution of 4,7-diiodobenzo[c][1,2,5]thiadiazole (388 mg, 1.0 mmol) and Pd(P(*t*-Bu)_3_)_2_ (24.6 mg, 0.050 mmol) in dry toluene (20 mL) and the mixture was stirred for 8 days at 0 °C. The reaction mixture was quenched with water and the organic layer was separated. The aqueous layer was extracted with CH_2_Cl_2_ three times. The combined organic layer was washed with brine and dried over sodium sulfate. The solvent was evaporated under reduced pressure, partially purified by column chromatography on silica gel (eluent: hexane/ethyl acetate = 25/1), and completely purified by recycling HPLC with a GPC column (eluent: CHCl_3_/Et_3_N = 99.5/0.5) to afford **1** as a pale yellow solid in 29% yield (186 mg).

### 3.6. Characterization Data for ***1***–***5***

*4,7-Bis(2-(2,4-dimethoxyphenyl)-1,1,2,2-tetramethyldisilaneyl)benzo[c][1,2,5]thiadiazole* (**1**). Yield: 29%. Colorless powder. Mp: 90.1–92.2 °C. ^1^H-NMR (500 MHz, CDCl_3_) δ 7.48 (s, 2H), 7.13 (d, 2H, *J* = 8.2 Hz), 6.39 (dd, 2H, *J* = 2.2, 8.2 Hz), 6.22 (d, *J* = 2.2 Hz, 2H), 3.76 (s, 6H), 3.51 (s, 6H), 0.44 (s, 12H), 0.24 (s, 12H). ^13^C-NMR (125 MHz, CDCl_3_) δ 164.8 (C_q_), 162.1 (C_q_), 158.0 (C_q_), 135.5 (CH), 135.4 (C_q_), 134.6 (CH), 118.3 (C_q_), 104.3 (CH), 97.0 (CH), 55.1 (CH_3_), 54.4 (CH_3_), −3.2 (CH_3_), −3.4 (CH_3_). FAB–LRMS *m*/*z* 640 (M^+^). FAB–HRMS Calcd for C_30_H_44_O_4_N_2_SSi_4_: 640.2099, Found: 640.2112 (M^+^).

*4,7-Bis(1,1,2,2-tetramethyl-2-(thiophen-2-yl)disilaneyl)benzo[c][1,2,5]thiadiazole* (**2**). Yield: 26%. Colorless powder. Mp: 61.4–63.1 °C. ^1^H-NMR (500 MHz, CDCl_3_) δ 7.55 (s, 2H), 7.51 (d, 2H, *J* = 4.5 Hz), 7.10–7.08 (m, 4H), 0.52 (s, 12H), 0.39 (s, 12H). ^13^C-NMR (125 MHz, CDCl_3_) δ 158.0 (C_q_), 138.5 (C_q_), 135.3 (CH), 134.3 (C_q_), 134.2 (CH), 130.4 (CH), 128.0 (CH), −2.0 (CH_3_), −3.7 (CH_3_). FAB–LRMS *m*/*z* 532 (M^+^). FAB–HRMS Calcd for C_22_H32N2S3Si4: 532.0805, Found: 532.0792 (M^+^).

*5,7-Bis(2-(2,4-dimethoxyphenyl)-1,1,2,2-tetramethyldisilaneyl)-2,3-dimethylthieno[3,4-b]pyrazine* (**3**). Yield: 8%. Colorless powder. Mp: 87.1–89.0 °C. ^1^H-NMR (500 MHz, CDCl_3_) δ 7.18 (d, 2H, *J* = 8.2 Hz), 6.42 (dd, 2H, *J* = 2.0, 8.0 Hz), 6.29 (d, *J* = 2.2 Hz, 2H), 3.78 (s, 6H), 3.61 (s, 6H), 2.51 (s, 6H), 0.45 (s, 12H), 0.28 (s, 12H). ^13^C-NMR (125 MHz, CDCl_3_) δ 165.0 (C_q_), 162.1 (C_q_), 150.1 (C_q_), 149.6 (C_q_), 136.4 (C_q_), 135.8 (CH), 118.5 (C_q_), 104.3 (CH), 97.0 (CH), 55.1 (CH_3_), 54.5 (CH_3_), 23.4 (CH_3_), −2.5 (CH_3_), −3.2 (CH_3_). FAB–LRMS *m*/*z* 668 (M^+^). FAB–HRMS Calcd for C_32_H_48_O_4_N_4_SSi_4_: 668.2412, Found: 668.2393 (M^+^).

*2,3-Dimethyl-5,7-bis(1,1,2,2-tetramethyl-2-(thiophen-2-yl)disilaneyl)thieno[3,4-b]pyrazine* (**4**). Yield: 46%. Yellow oil. ^1^H-NMR (500 MHz, CDCl_3_) δ 7.56 (dd, 2H, *J* = 1.1, 4.6 Hz), 7.19–7.15 (m, 4H), 2.57 (s, 6H), 0.51 (s, 12H), 0.42 (s, 12H). ^13^C-NMR (125 MHz, CDCl_3_) δ 151.0 (C_q_), 150.1 (C_q_), 138.7 (C_q_), 135.7 (C_q_), 134.4 (CH), 130.5 (CH), 128.2 (CH), 23.5 (CH_3_), −1.9 (CH_3_), −2.7 (CH_3_). FAB–LRMS *m*/*z* 560 (M^+^). FAB–HRMS Calcd for C_24_H_36_N_2_S_3_Si_4_: 560.1118, Found: 560.1108 (M^+^).

*7,7′-Bis(2-(4-methoxyphenyl)-1,1,2,2-tetramethyldisilaneyl)-4,4′-bibenzo[c][1,2,5]thiadiazole* (**5**). Yield: 36%. Yellow oil. ^1^H-NMR (500 MHz, CDCl_3_) δ 8.23 (d, 2H, *J* = 6.9 Hz), 7.57 (d, 2H, *J* = 4.3 Hz), 7.31 (d, 4H, *J* = 8.5 Hz), 6.82 (d, 4H, *J* = 8.5 Hz), 3.78 (s, 6H), 0.52 (s, 12H), 0.36 (s, 12H). ^13^C-NMR (125 MHz, CDCl_3_) δ 160.0 (C_q_), 159.4 (C_q_), 152.9 (C_q_), 135.7 (CH), 135.3 (C_q_), 135.1 (CH), 130.3 (CH), 130.0 (C_q_), 129.6 (C_q_), 113.5 (CH), 55.0 (CH_3_), −3.1 (CH_3_), −3.5 (CH_3_). FAB–LRMS *m*/*z* 714 (M^+^). FAB–HRMS Calcd for C_34_H_42_O_2_N_4_S_2_Si_4_: 714.1826, Found: 714.1819 (M^+^).

## 4. Conclusions

Disilane-linked D/A/D compounds **1**–**5** were designed and synthesized by Pd-catalyzed coupling of aryl iodides with hydrosilane precursors. The compounds were characterized by NMR spectroscopy, HRMS, and UV-vis absorption and fluorescence spectroscopy. XRD analysis of **1** and computational calculations of **1**–**5** furthered our understanding of the optical properties of these compounds and revealed several features underlying the relationship between the structure and the photophysical properties.

The substituent at the *o*-position against to Si‒Si unit of the donor moiety contrastively affected the photoluminescence properties of disilane-linked D/A/D molecules in solution and in the solid state. Molecules with a substituent at the *o*-position may suppress intramolecular motion such as internal rotation, leading to non-radiative decay, resulting in increased emission intensity in solution. However, in the solid state, the substituent at the *o*-position produces a twisted conformation and reduces intramolecular interaction between the donor and acceptor moieties, leading to decreased emission intensity. Compounds with no substituent at the *o*-position showed low emission intensity in the solution state, whereas their emission intensity was much higher in the solid state. Accordingly, compound **2** exhibited a high solid-state fluorescence quantum yield that was nearly two orders of magnitude larger than that in solution. These results indicate that substituents increasing emission efficiency in solution are not necessarily effective even in the solid state. In addition, these results are important findings in the molecular design of disilane-linked molecules whose reported examples are still limited.

## Figures and Tables

**Table 1:** Photophysical Properties of Compounds **1**–**5**.

Compound	In CH_2_Cl_2_^a^							In the Solid State				
	*λ*_abs_ (nm) ^b^	*ε* (M^−1^ cm^−1^) ^c^	*λ*_em_ (nm) ^d^	*Φ*_F_^e^	*τ* (ns) ^f^	*k_f_* (ns^−1^) ^g^	*k_nr_* (ns^−1^) ^h^	*λ*_em_ (nm) ^i^	*Φ*_F_^e^	*τ* (ns) ^f^	*k_f_* (ns^−1^) ^g^	*k_nr_* (ns^−1^) ^h^
**1**	360	7960	487	0.004	− ^j^	− ^j^	− ^j^	536	0.006	0.47	0.013	2.1
**2**	353	7670	497	0.002	− ^j^	− ^j^	− ^j^	490	0.167	2.8	0.060	0.30
**3**	385	4280	503	0.097	2.8	0.035	0.32	456	0.019	0.91	0.021	1.1
**4**	380	5280	495	0.021	0.58	0.034	1.7	− ^k^	− ^k^	− ^k^	− ^k^	− ^k^
**5**	388	9960	460	0.023	0.69	0.033	1.4	− ^k^	− ^k^	− ^k^	− ^k^	− ^k^

^a^ Measured in anhydrous degassed CH_2_Cl_2_. ^b^ Only the longest maxima are shown. ^c^ Molar extinction coefficient. ^d^ Excited at the longest absorption maximum. ^e^ Absolute quantum yields determined using an integrating sphere system. ^f^ Fluorescence lifetime detected at the maximum fluorescence wavelength. ^g^ Fluorescence rate constant, *k*_f_ = *Φ*_F_/*τ*. ^h^ Non-radiative rate constant, *k*_nr_ = (1 – *Φ*_F_)/*τ*. ^i^ Compounds **1**, **2** and **3** were excited at 373, 436, and 429 nm, respectively. Excitation wavelengths in the solid state were determined from the excitation spectra. ^j^ Fluorescence below the detection limit. ^k^ Product obtained as an oil.

## Supplementary Materials

Crystallographic data for **1**, theoretical calculations for **1**–**5**, and copies of the ^1^H & ^13^C-NMR spectra of **1**–**5** are available online.
