# Supplementary material for: Effects of Substituents on the Blue Luminescence of Disilane-Linked Donor‒Acceptor‒Donor Triads
Source: Molecules. 2019 Jan 31;24(3):521. doi: 10.3390/molecules24030521 (PMC6384591; doi:10.3390/molecules24030521)
Supplement: Supplementary file 1 [file molecules-24-00521-s001.pdf]

Supplementary Material

for

Effects of substituents on the blue luminescence of disilane-linked donor–acceptor–donor triads

Tsukasa Usuki [1], Kenichiro Omoto [1], Masaki Shimada [1], Yoshinori Yamanoi [1,*], Hidetaka Kasai [2], Eiji Nishibori [2], and Hiroshi Nishihara [1,*]

[1] Department of Chemistry, School of Science, The University of Tokyo, 7-3-1 Hongo, Bunkyo-ku, Tokyo 113-0033, Japan

[2] Division of Physics, Faculty of Pure and Applied Sciences, Tsukuba Research Center for Interdisciplinary Materials Science (TIMS), and Center for Integrated Research in Fundamental Science and Engineering (CiRfSE), University of Tsukuba, 1-1-1 Tennodai, Tsukuba, Ibaraki 305-8571, Japan

Contents

1. Crystallographic data of 1 .... S2

2. Powder X-ray diffraction analysis of 2 .... S5

3. Theoretical calculation of 1–5 .... S6

4. Copies of the ^{1}H & ^{13}C NMR spectra of 1–5 .... S9

1. Crystallographic data of 1

Table S1. Crystallographic data of **1**

Empirical formula	$C_{30}H_{44}N_2O_4SSi_4$
Fw / g mol^{-1}	641.09
Crystal system	triclinic
Space group	P-1 *(#2)*
Crystal size / mm	0.1 × 0.1 × 0.1
Temperature / K	93
a / Å	10.029(3)
b / Å	13.257(4)
c / Å	14.692(5)
α / °	87.77(1)
β / °	71.652(9)
γ / °	73.114(9)
V / Å^3	1771.0(9)
Z	2
D_{calcd} g/cm^{-3}	1.202
λ / Å	0.71075
μ / mm^{-1}	0.261
Reflections collected	13221
Independent reflections	7099
$F_{(000)}$	684.00
R_{int}	0.0638
R_1 ($I > 2.00s(I)$)^a	0.0579
wR_2 (All reflections)^b	0.1617
GoF^c	1.034

^{a} $R_1 = \Sigma||F\mathrm{o}| - |Fc||/\Sigma|F\mathrm{o}|$ ($I > 2\ s(I)$). ^{b} $wR_2 = [\Sigma(w(F\mathrm{o}^2 - F\mathrm{c}^2)^2/\Sigma w(F\mathrm{o}^2)^2]^{1/2}$ ($I > 2\ s(I)$). ^{c} GOF = $[\Sigma(w(F\mathrm{o}^2 - F\mathrm{c}^2)^2/\Sigma(N\mathrm{r} - N\mathrm{p})^2]$s

Table S2. Selected bond lengths, angles, and torsion angles of **1**

**Bond lengths (Å)**	
Si1-Si2	2.349(1)
Si1-C21	1.867(3)
Si1-C22	1.871(4)
Si1-C27	1.882(3)
Si2-C16	1.892(3)
Si2-C19	1.870(3)
Si2-C20	1.876(4)
Si3-Si4	2.355(1)
Si3-C11	1.872(3)
Si3-C12	1.876(4)
Si3-C13	1.895(3)
Si4-C5	1.883(3)
Si4-C9	1.875(4)
Si4-C10	1.872(3)
**Angles (º)**	
Si1-Si2-C16	110.5(1)
Si2-Si1-C27	108.8(1)
Si3-Si4-C5	109.6(1)
Si4-Si3-C13	111.1(1)
**Torsion angles (º)**	
C27-Si1-Si2-C16	-135.1(1)
Si1-Si2-C16-C15	124.2(2)
Si1-Si2-C16-C17	-56.1(2)
Si2-Si1-C27-C26	120.3(2)
Si2-Si1-C27-C28	-60.3(2)
C13-Si3-Si4-C5	-133.2(1)
Si3-Si4-C5-C4	131.4(2)
Si3-Si4-C5-C6	-49.9(3)

Si4-Si3-C13-C14	127.4(2)
Si4-Si3-C13-C18	-52.6(2)

2. Powder X-ray diffraction analysis of 2

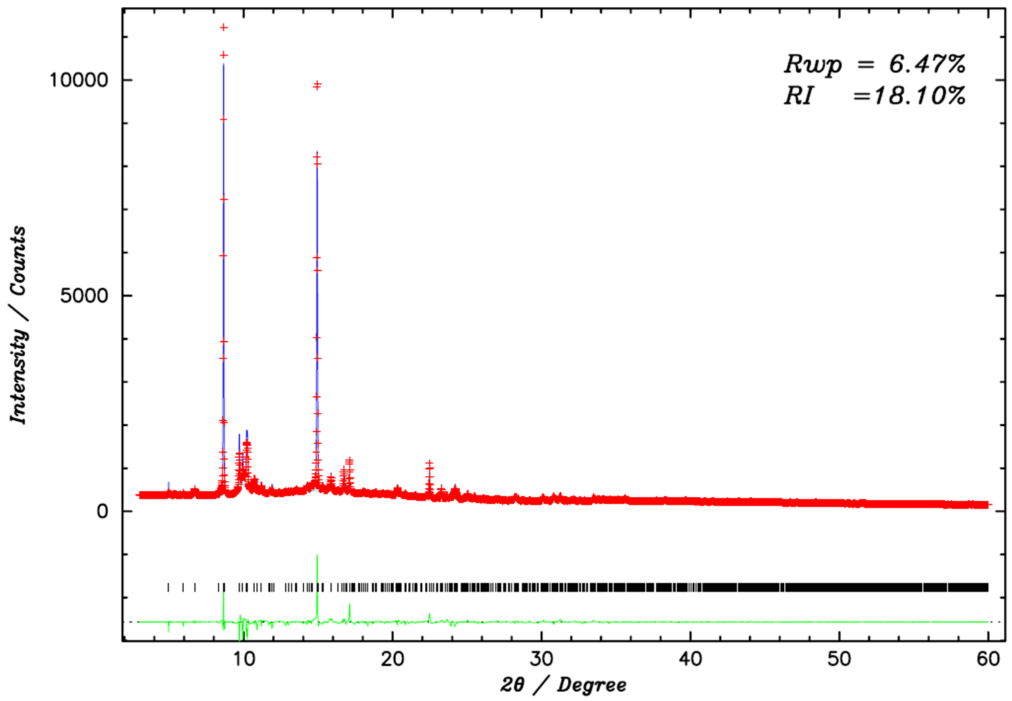

Figure S1. Fitting result of Rietveld refinement for **2**. The red corss is observed data, the blue line is the calculated profile, green line is the difference between observed and calculated profile. The black bars indicate the position of bragg peaks.

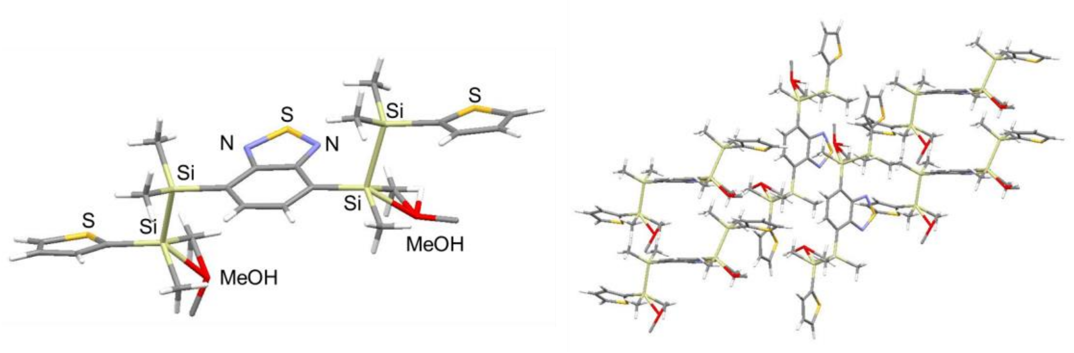

Figure S2. Plausible crystal structure of **2** (left) and packing structure of **2** (right) based on Rietveld refinement. Hydrogen atoms of solvent molecules (MeOH molecules) are not assigned.

3. Theoretical calculation of 1–5

(a) Frontier molecular orbitals and energy level

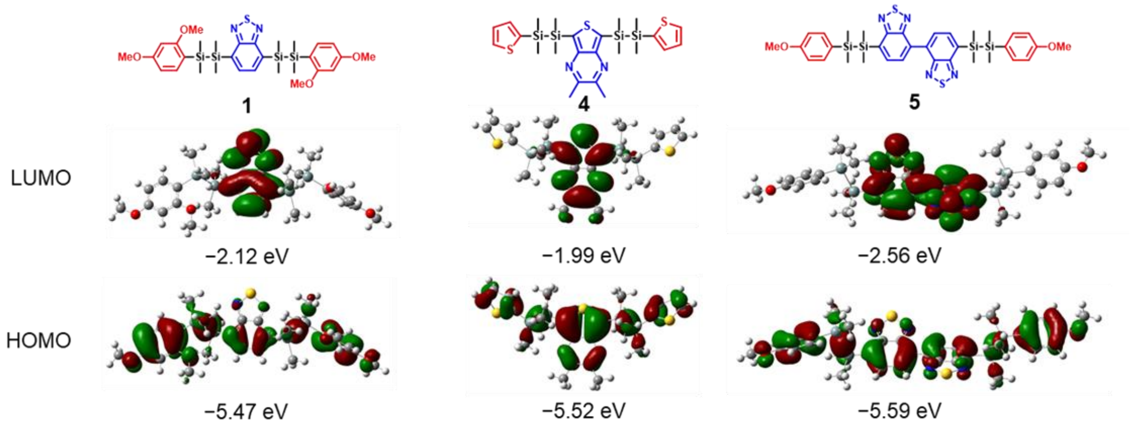

Figure S3. Frontier molecular orbitals (an absolute isovalue *l* = 0.02) and energy levels for **1**, **4**, and **5** calculated with DFT at the B3LYP/6-31G (d, p) level of theory.

(b) TD-DFT calculation

f: Oscillator strength. H: HOMO. L: LUMO. *c*: CI expansion coefficients for each excitation.

Table 3. Summary of the TD-DFT calculation of **1**.

S_1: 428.10 nm			$f = 0.0990$
H	→	L	*c*: 0.69803
S_2: 408.06 nm			$f = 0.0019$
H–1	→	L	*c*: 0.70331
S_3: 359.81 nm			$f = 0.0763$
H–2	→	L	*c*: 0.69342

Table 4. Summary of the TD-DFT calculation of **2**.

S_1: 417.24 nm			$f = 0.1394$
H	→	L	*c*: 0.70166
S_2: 386.72 nm			$f = 0.0001$
H–1	→	L	*c*: 0.70287
S_3: 336.68 nm			$f = 0.0584$
H–2	→	L	*c*: 0.69655

Table 5. Summary of the TD-DFT calculation of **3**.

S_1: 413.79 nm			$f = 0.1593$
H	→	L	*c*: 0.69626
S_2: 368.12 nm			$f = 0.0061$
H–1	→	L	*c*: 0.68856
S_3: 367.31 nm			$f = 0.0059$
H-5	→	L	*c*: 0.63761

Table 6. Summary of the TD-DFT calculation of **4**.

S_1: 408.01 nm			f = 0.1678
H	→	L	c: 0.69582
S_2: 368.40 nm			f = 0.0063
H–3	→	L	c: 0.64557
S_3: 350.61 nm			f = 0.0077
H-1	→	L	c: 0.70126

Table 7. Summary of the TD-DFT calculation of **5**.

S_1: 470.60 nm			f = 0.4150
H	→	L	c: 0.69596
S_2: 440.00 nm			f = 0.0033
H–1	→	L	c: 0.68431
S_3: 416.90 nm			f = 0.0000
H	→	L+1	c: 0.64947

3. Copies of ^1H and ^{13}C NMR of 1–5

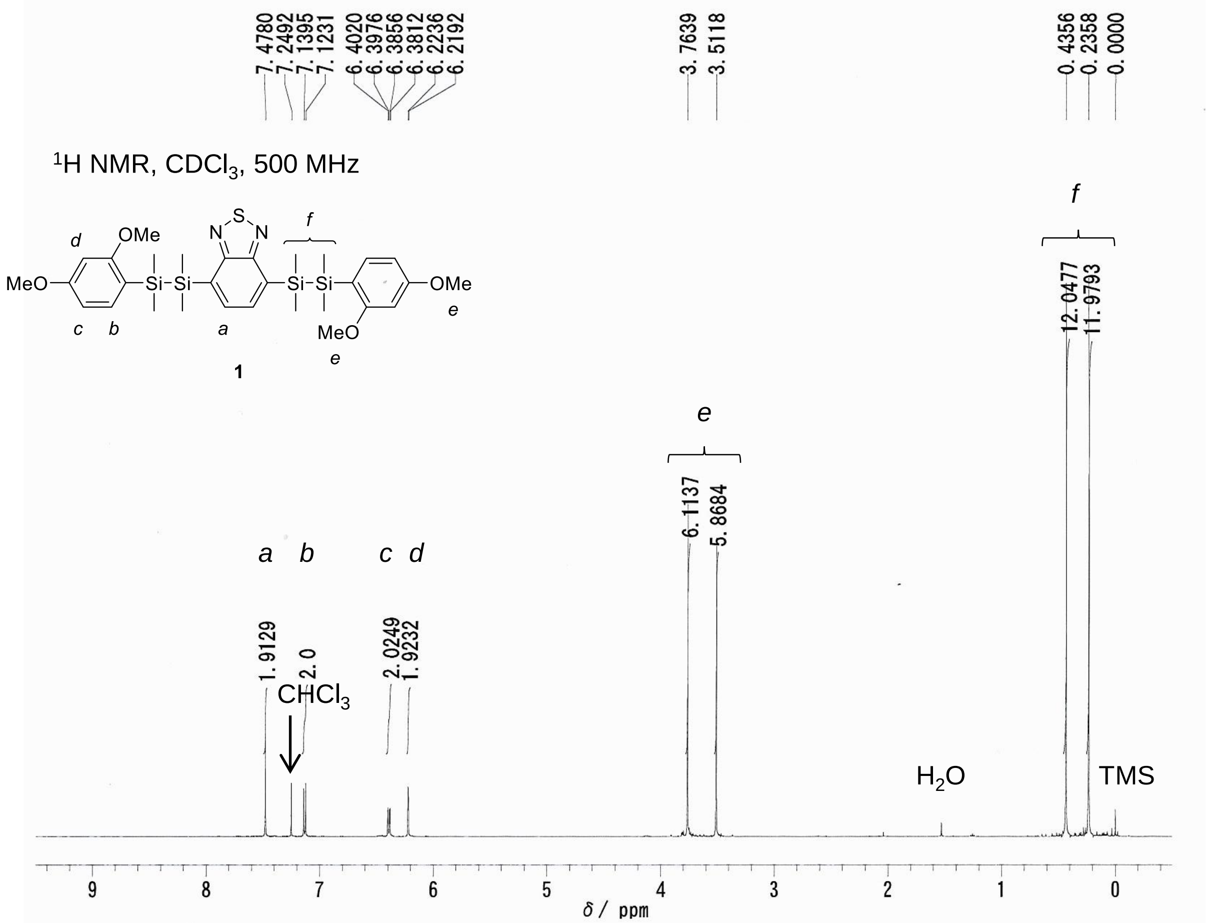

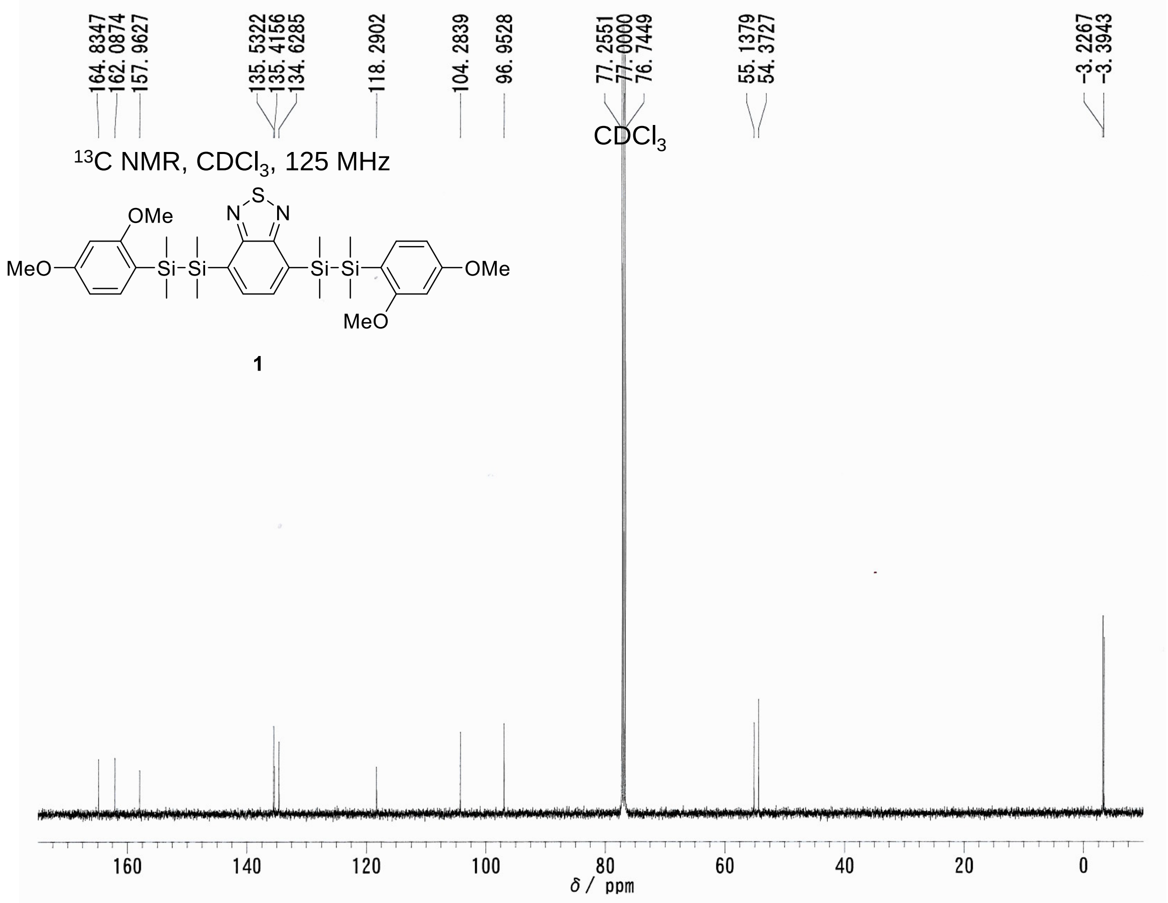

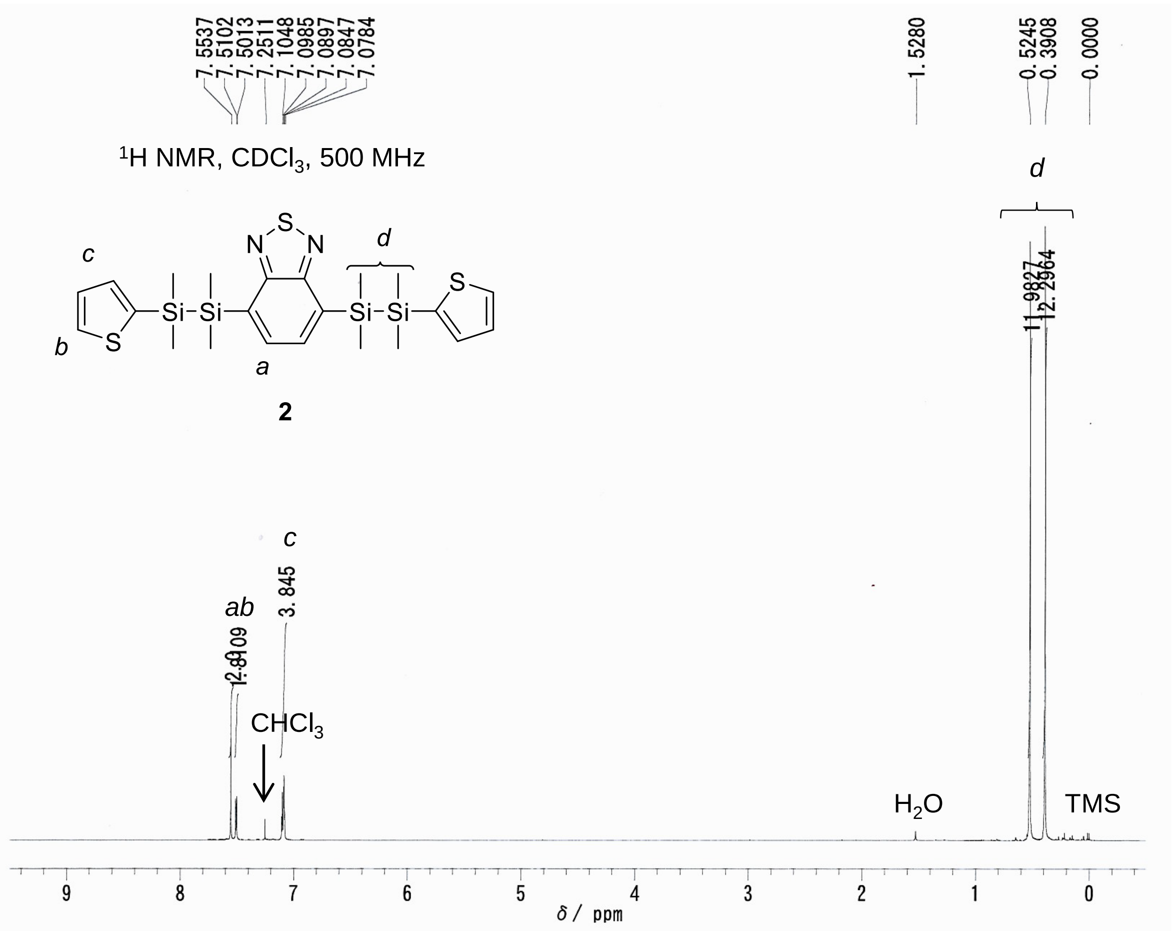

7.5537
7.5102
7.5013
7.2511
7.1048
7.0985
7.0897
7.0847
7.0784
1.5280
0.5245
0.3908
0.0000
1H NMR, CDCl3, 500 MHz
d
c
b
a
2
11.9827
12.2964
ab
c
3.845
CHCl3
H2O
TMS
δ / ppm

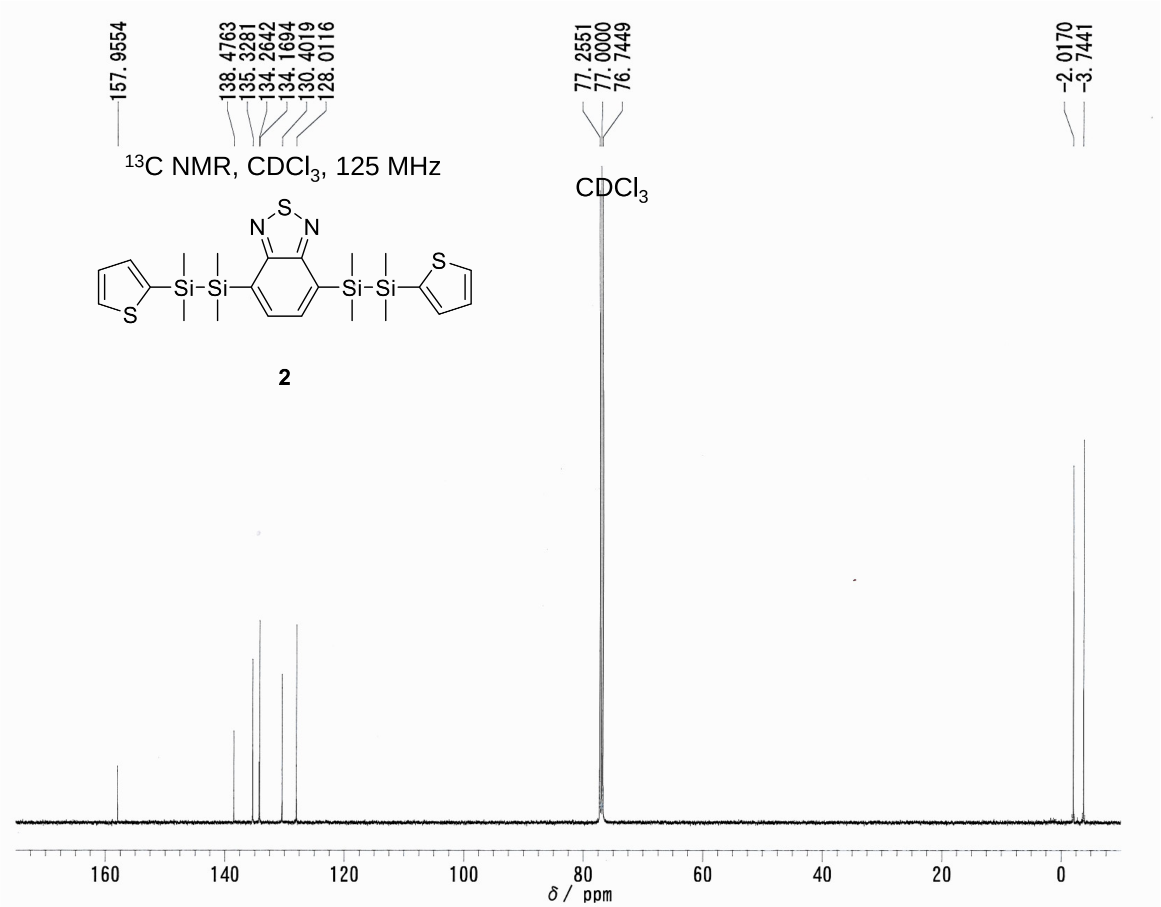

157.9554
138.4763
135.3281
134.2642
134.1694
130.4019
128.0116
77.2551
77.0000
76.7449
−2.0170
−3.7441
13C NMR, CDCl3, 125 MHz
CDCl3
2
δ / ppm

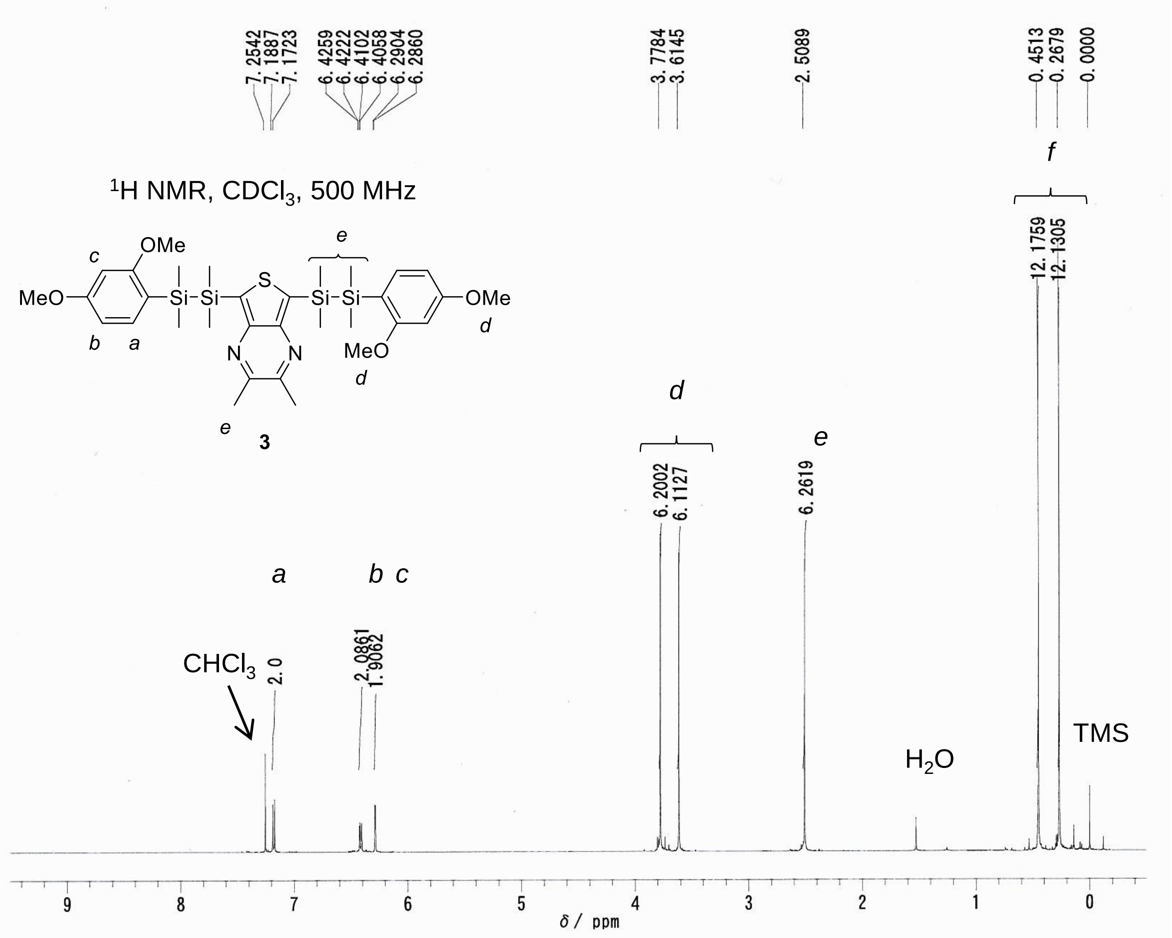

7.2542
7.1887
7.1723
6.4259
6.4222
6.4102
6.4058
6.2904
6.2860
3.7784
3.6145
2.5089
0.4513
0.2679
0.0000
f
12.1759
12.1305
1H NMR, CDCl3, 500 MHz
OMe
c
e
MeO
Si
Si
S
Si
Si
OMe
d
b
a
N
N
MeO
d
e
3
d
6.2002
6.1127
e
6.2619
a
b c
CHCl3
2.0
2.0861
1.9062
TMS
H2O
9
8
7
6
5
4
3
2
1
0
δ / ppm

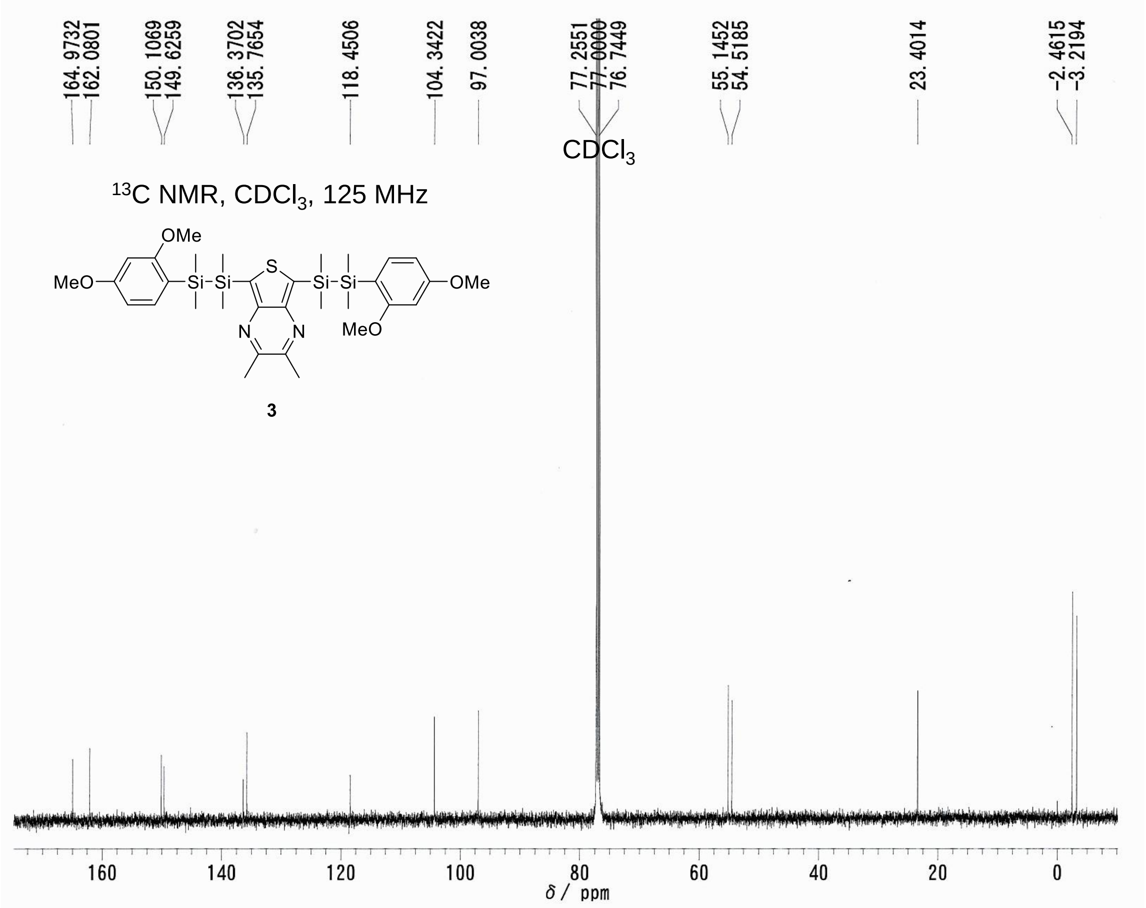

164.9732
162.0801
150.1069
149.6259
136.3702
135.7654
118.4506
104.3422
97.0038
77.2551
77.0000
76.7449
55.1452
54.5185
23.4014
−2.4615
−3.2194
CDCl3
13C NMR, CDCl3, 125 MHz
OMe
MeO
Si
Si
S
Si
Si
OMe
N
N
MeO
3
160
140
120
100
80
60
40
20
0
δ / ppm

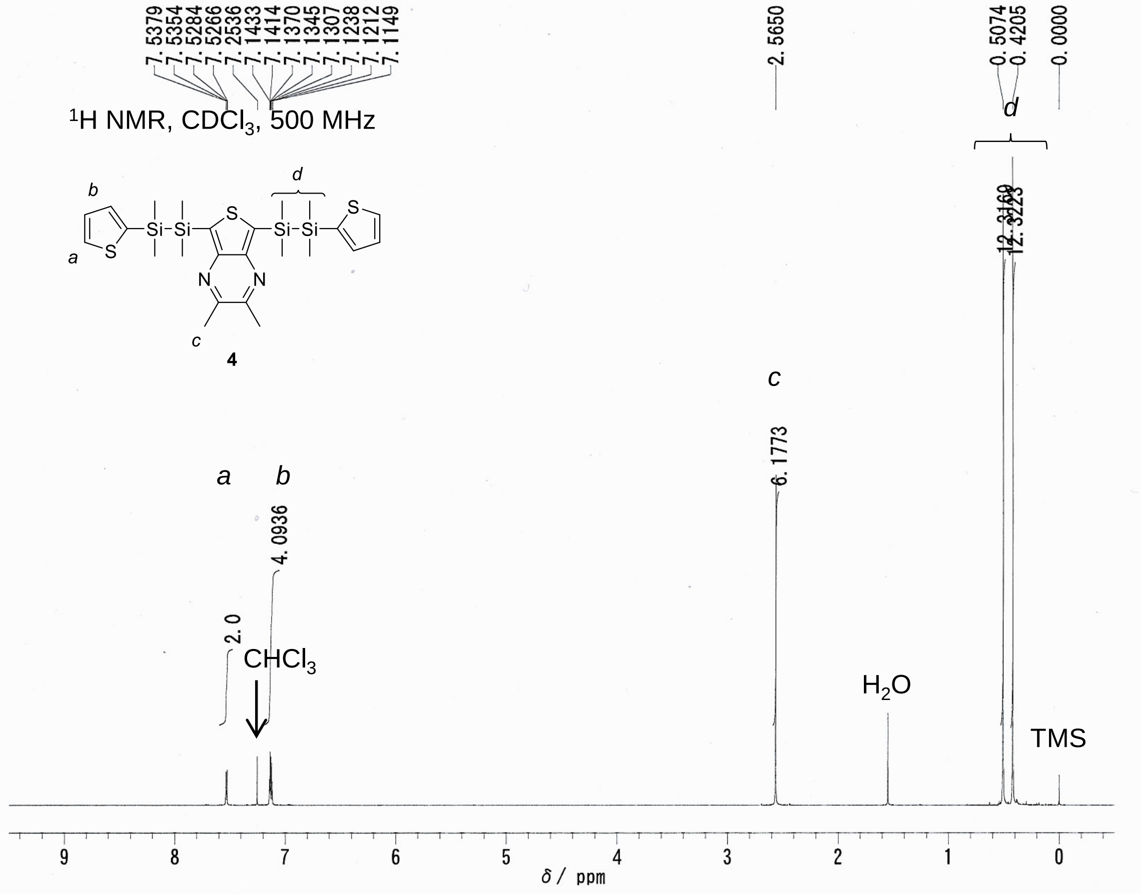
7.5379
7.5354
7.5284
7.5266
7.2536
7.1433
7.1414
7.1370
7.1345
7.1307
7.1238
7.1212
7.1149
2.5650
0.5074
0.4205
0.0000
1H NMR, CDCl3, 500 MHz
a
b
c
d
4
2.0
4.0936
6.1773
12.3160
12.3223
CHCl3
H2O
TMS
δ / ppm

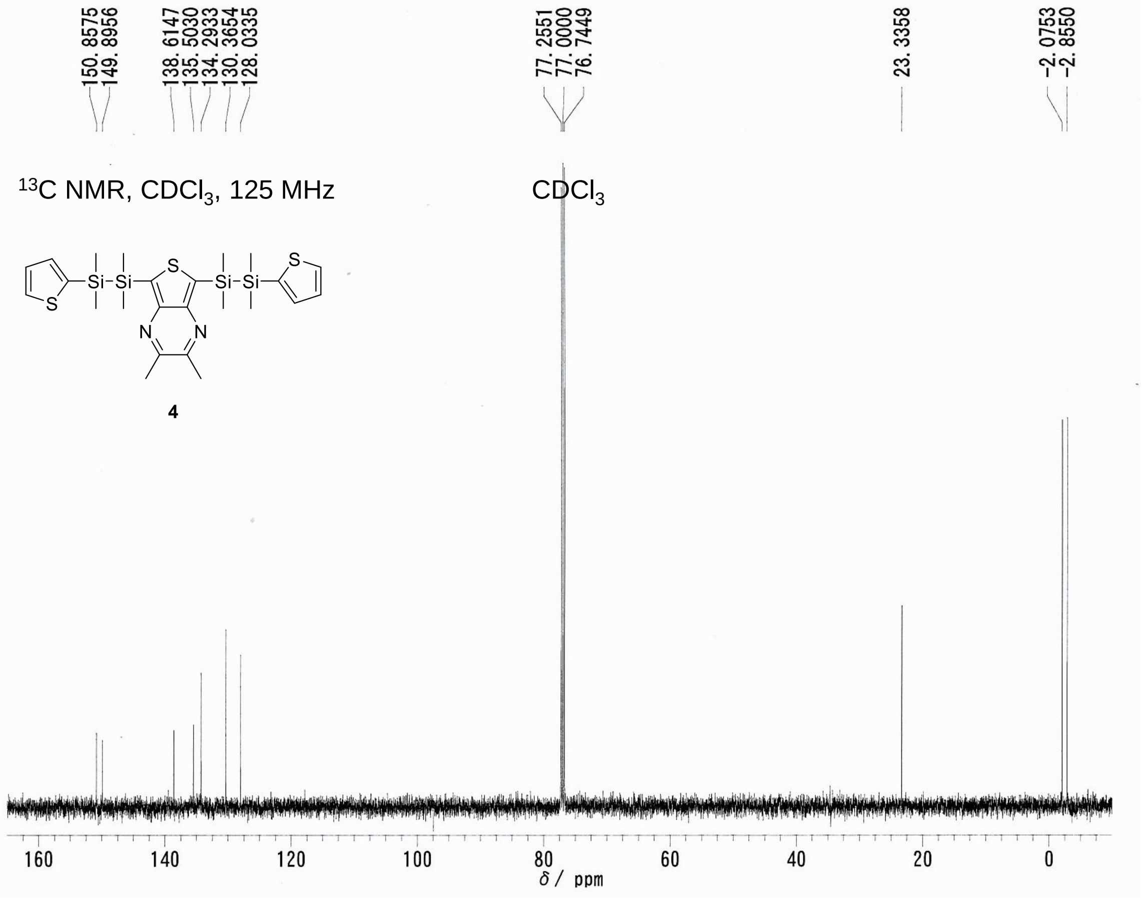
150.8575
149.8956
138.6147
135.5030
134.2933
130.3654
128.0335
77.2551
77.0000
76.7449
23.3358
-2.0753
-2.8550
13C NMR, CDCl3, 125 MHz
CDCl3
4
δ / ppm

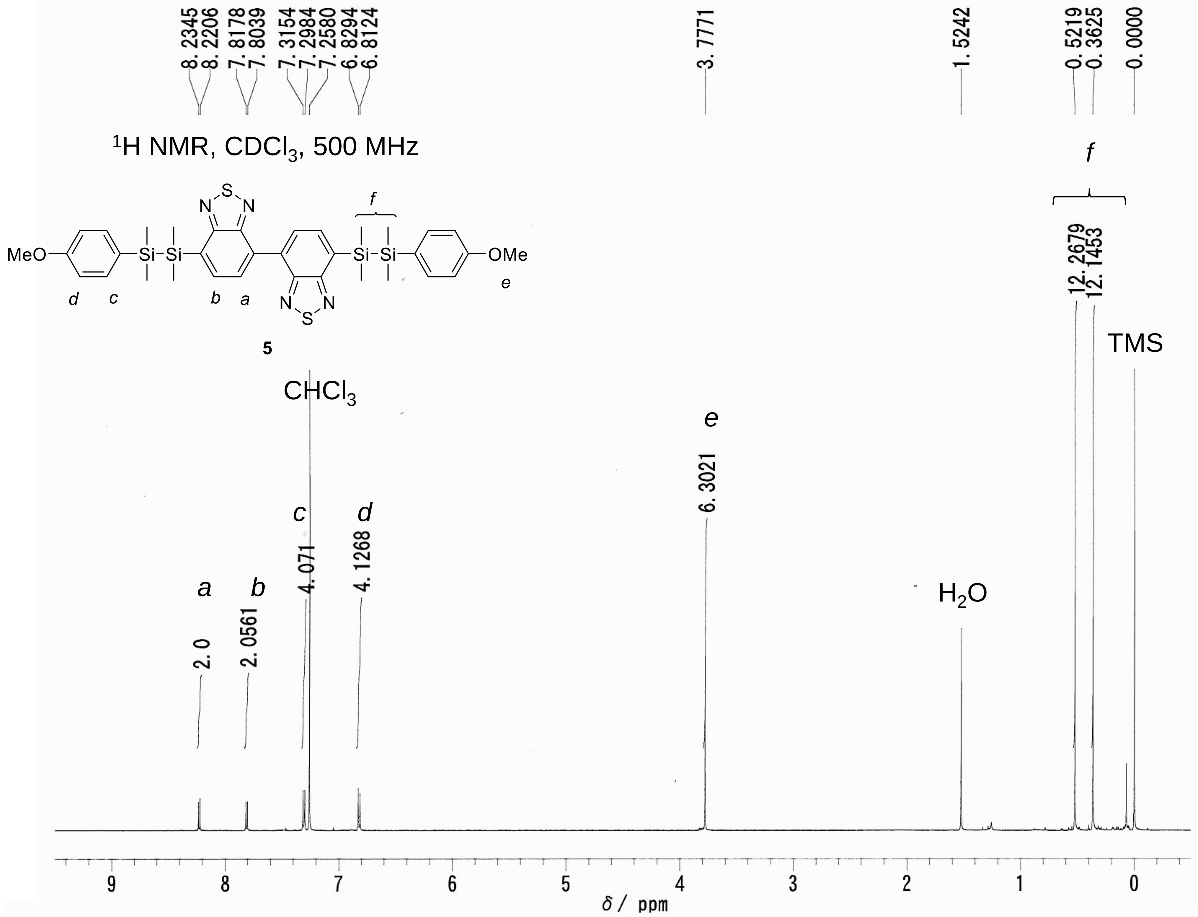
8.2345
8.2206
7.8178
7.8039
7.3154
7.2984
7.2580
6.8294
6.8124
3.7771
1.5242
0.5219
0.3625
0.0000
1H NMR, CDCl3, 500 MHz
f
MeO
Si
Si
N
S
N
OMe
d c b a e
5
12.2679
12.1453
TMS
CHCl3
e
6.3021
c d
4.071
4.1268
a b
2.0
2.0561
H2O
9 8 7 6 5 4 3 2 1 0
δ / ppm

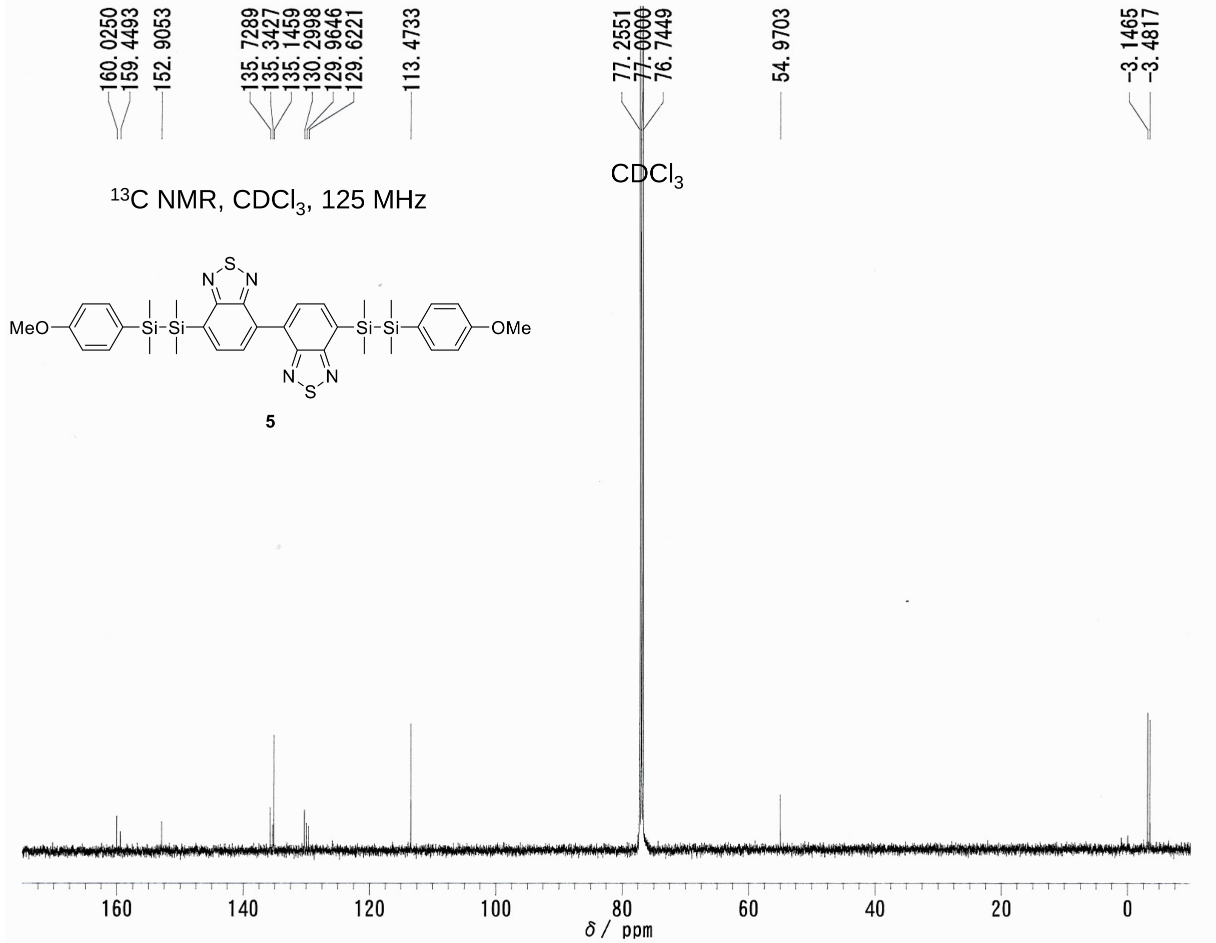
160.0250
159.4493
152.9053
135.7289
135.3427
135.1459
130.2998
129.9646
129.6221
113.4733
77.2551
77.0000
76.7449
54.9703
−3.1465
−3.4817
CDCl3
13C NMR, CDCl3, 125 MHz
MeO
Si
Si
N
S
N
OMe
5
160 140 120 100 80 60 40 20 0
δ / ppm
